# Trypsin-Ligand binding affinities calculated using an effective interaction entropy method under polarized force field

**DOI:** 10.1038/s41598-017-17868-z

**Published:** 2017-12-18

**Authors:** Yalong Cong, Mengxin Li, Guoqiang Feng, Yuchen Li, Xianwei Wang, Lili. Duan

**Affiliations:** 1 Shandong Province Key Laboratory of Medical Physics and Image Processing Technology, School of Physics and Electronics, Shandong Normal University, Jinan, 250014 China; 2 Center for Optics & Optoelectronics Research, College of Science, Zhejiang University of Technology, Hangzhou, 310023 China

## Abstract

Molecular dynamics (MD) simulation in the explicit water is performed to study the interaction mechanism of trypsin-ligand binding under the AMBER force field and polarized protein-specific charge (PPC) force field combined the new developed highly efficient interaction entropy (IE) method for calculation of entropy change. And the detailed analysis and comparison of the results of MD simulation for two trypsin-ligand systems show that the root-mean-square deviation (RMSD) of backbone atoms, B-factor, intra-protein and protein-ligand hydrogen bonds are more stable under PPC force field than AMBER force field. Our results demonstrate that the IE method is superior than the traditional normal mode (Nmode) method in the calculation of entropy change and the calculated binding free energy under the PPC force field combined with the IE method is more close to the experimental value than other three combinations (AMBER-Nmode, AMBER-IE and PPC-Nmode). And three critical hydrogen bonds between trypsin and ligand are broken under AMBER force field. However, they are well preserved under PPC force field. Detailed binding interactions of ligands with trypsin are further analyzed. The present work demonstrates that the polarized force field combined the highly efficient IE method is critical in MD simulation and free energy calculation.

## Introduction

Underlying the interaction mechanism of protein-ligand at atomic level is vital in biomolecular and can provide extremely high value in drug design. Molecular dynamics (MD)^1^ simulation is the most commonly used and most valuable tool in studying the binding of protein and ligand. The accuracy of the results in MD simulation mainly depends on the molecular force field used. The current force fields, such as AMBER, CHARMM, GROMOS, OPLS and so on, lack the electronic polarization effect^2,3^ which lead inaccurate and unreliable results. In these force fields, those charges of residues in proteins are fixed despite of the different surroundings. As the result, they fail to give the accurate representations of the electrostatic environment. Extensive studies have found electronic interaction plays an essential role in many properties of biomolecules.

To provide a more reliable description of the electronic interaction for the binding between protein and ligand, we employ the polarized protein-specific charge (PPC) force^4–6^ field derived from quantum mechanical calculation for protein and ligand using the molecular fractionation with conjugate caps approach^7^. PPC can provide accurate electrostatic interactions for protein and extensive works have demonstrated that the electronic polarization effect has a significant impact on the structure and function of protein^8–14^.

The binding free energy is used to determine the strength of the binding between protein and ligand and accurate prediction of the binding free energy is very important. So far, several methods are used to calculate the binding free energy, in which the most accurate and rigorous methods are free energy perturbation (FEP)^15–19^ and thermodynamic integration(TI)^20,21^. However, the above methods are extremely expensive and time-consuming. Besides, they can only calculate the relative binding free energy^22^, so that the application of these two methods in drug design has been greatly limited. In contrast, the Molecular Mechanics/Poisson-Boltzmann Surface Area (MM/PBSA)^23–26^ approach is more convenient in computing binding free energy. It is worth mentioned that this method is faster by several orders of magnitude than FEP and TI methods^27^. Therefore, the computational cost of this method is low. However, the method of MM/PBSA has a major problem that the entropy contribution is calculated by the standard normal mode (Nmode) method which is time-consuming and approximate. As a result, the binding free energy calculated by the MM/PBSA method is uncertain and unreliable.

In current, many methods have been developed to calculate entropy. For example, there is an empirical method to calculate the entropy^28,29^. This method divides entropy contribution into two parts: solvation free entropy and conformational free entropy. The solvation free entropy can be calculated with heat capacity. The conformational free entropy has relation with the number of rotatable bonds compared with other methods. In this report, we employ a new method called interaction entropy^30^ (IE) to compute the entropy change which is more theoretically rigorous, more computationally efficient and more time-saving. The interaction energy contribution can be obtained directly from the MD simulation without any additional computational time. As a result, the solvation free energy is obtained by the PBSA module in MM/PBSA method and the entropy contribution is calculated by IE method during the calculation of the binding free energy.

Understanding the binding mechanism between trypsin and its ligand can provide useful information for developing novel trypsin inhibitor. Trypsin is a kind of protease^31^ that acts as a digestive enzyme in vertebrates, playing an important role in the digestion of proteins in the small intestine. Trypsin acts as a typical serine protease, which cleaves peptide chains mainly at the carboxyl side of the amino acids lysine or arginine by using a special serine amino acid, playing a vital role in physiological functions. In the current, trypsin inhibitors are classified two kinds. One is small protein and the other is polypeptide that can inhibit activity of trypsin. Because of its particular physiological properties, trypsin inhibitor has attracted more attention. Paulius Mikulskis *et al*.^32^, have studied the binding affinities between 34 ligands and trypsin by using MM/PBSA, MM/GBSA, LIE, continuum LIE and Glide score methods based on MD stimulation. They found these methods were failed to give an accurate result. Up to now, there have been considerable researches on ligands targeting trypsin, but most researches ignored the effect of the polarization, leading inaccurate results of the binding free energy. Especially, the study about the binding affinity of trypsin and ligand is scarce using PPC force field combined the IE method.

In this report, MD simulations are performed under AMBER and PPC force field to calculate the binding free energy between trypsin and ligands, respectively. The solvation free energy is calculated by standard MM/PBSA method. The entropy contribution is calculated by Nmode and IE methods. The calculated binding free energy of two trypsin systems are analyzed and compared, and our results show that PPC force field with IE method is the most optimal combination in MD simulation and free energy calculation for our systems.

## Method

### Polarized Protein-Specific Charge

PPC^33–35^ which derived from quantum calculation is employed to provide accurate partial atomic charges of proteins to represent electrostatic polarization effect. First, the method of molecular fragmentation using a conjugated caps scheme (MFCC)^7^ cleaves the protein into fragments at the peptide bond and adds a pair of conjugate caps at both ends of the fragment to achieve the electronic structure of the protein through fully quantum mechanical (QM) calculation. Then, the RESP^36^ program is used to fit atomic charges of the whole protein based on the obtained electron density distribution of each fragmental molecular. Next electrostatic solvation energy and induced surface charge can be calculated through solving the Poisson-Boltzmann (PB)^37^ Equation. The newly obtained charges of other residues and solvent are regarded as background charges in the QM calculation for each fragment. Finally, the solute and solvent polarize each other until solvation energy and induced charges converge.

### MM/PBSA method

The Molecular Mechanics/Poisson-Boltzmann Surface Area (MM/PBSA)^38,39^ model is one of the most widely used methods to compute the binding free energy. In principle, simulations of three trajectories (separate protein, separate ligand and the complex) should be performed to calculate the binding free energy of protein-ligand. However, the method of triple system simulations is time-consuming and inefficient. Therefore, most researchers have used single system (only complex) simulations instead of triple system to calculate the binding free energy. The method of single system simulations regards the protein-ligand structure as a rigid body and doesn’t take the strain energy produced by the conformational changes into account^40,41^.

According to the MM/PBSA method, binding free energy (Δ*G*_*blind*_) can be simply defined by the following equations:

1 $${\rm{\Delta }}{G}_{bind}={G}_{complex}-({G}_{protein}+{G}_{ligand})$$

where *G*_*complex*_, *G*_*protein*_ and *G*_*ligand*_ represent the free energies of the complex, protein and ligand, respectively. In addition, the binding free energy (Δ*G*_*blind*_) consists of two parts:

2 $${\rm{\Delta }}{G}_{bind}={\rm{\Delta }}{G}_{gas}+{\rm{\Delta }}{G}_{sol}$$

where Δ*G*_*gas*_ and Δ*G*_*sol*_ represent the gas phase free energy and the solvation free energy, respectively. And gas phase free energy (Δ*G*_*gas*_) can be divided into two parts:

3 $${\rm{\Delta }}{G}_{gas}=\langle {E}_{pl}^{\mathrm{int}}\rangle -T{\rm{\Delta }}S$$

where $\langle {E}_{pl}^{\mathrm{int}}\rangle$ represents protein-ligand interaction including electrostatic and van der Waals (vdW) interactions, and −*T*Δ*S* represents the contribution of entropy. In the meanwhile, the solvation free energy (Δ*G*_*sol*_) also can be divided into two parts:

4 $${\rm{\Delta }}{G}_{sol}={\rm{\Delta }}{G}_{pb}+{\rm{\Delta }}{G}_{np}$$

where Δ*G*_*pb*_ and Δ*G*_*np*_ represent the polar and non-polar solvation free energy terms, respectively. The term Δ*G*_*pb*_ can be computed through the PB equation. In our works, the exterior and interior dielectric constants are set to 80 and 1, respectively. And term Δ*G*_*np*_ is based on following equation:

5 $${\rm{\Delta }}{G}_{np}=\gamma \cdot SASA+\beta $$

where *SASA* represents solvent-accessible surface area, and it can be calculated by MSMS^42^ program. In our works, the numerical values of *γ* and *β* are the standard values of 0.00542 kcal (mol Å^2^)^−1^ and 0.92 kcal mol^−1^, respectively.

Finally, the entropy contribution toward the binding free energy can be computed by the AMBER NMODE (Nmode) module^43^. However, considering that it would be extremely time-consuming and computationally expensive to calculate the entropy contribution, we only extract 10 snapshots from trajectory to calculate.

### Interaction entropy (IE) method

Compared with Nmode method, the calculation of entropy contribution is replaced by more precise and concise formula in the IE method^30,44,45^. It can be defined as the following term:

6 $$-T{\rm{\Delta }}S=KT\,\mathrm{ln}\,\langle {e}^{\beta {\rm{\Delta }}{E}_{pl}^{\mathrm{int}}}\rangle $$

where ${\rm{\Delta }}{E}_{pl}^{\mathrm{int}}$ represents the fluctuation of protein-ligand interaction energy around the average energy. It can be calculated by the following formula:

7 $${\rm{\Delta }}{E}_{pl}^{\mathrm{int}}={E}_{pl}^{\mathrm{int}}-\langle {E}_{pl}^{\mathrm{int}}\rangle $$

where $\langle {E}_{pl}^{\mathrm{int}}\rangle$ represents the average of protein-ligand interaction energy.

The high efficiency of IE method results from that $\langle {E}_{pl}^{\mathrm{int}}\rangle$ and $\langle {e}^{\beta {\rm{\Delta }}{E}_{pl}^{\mathrm{int}}}\rangle$ can be computed conveniently and efficiently by the following equations:

8 $$\langle {E}_{pl}^{\mathrm{int}}\rangle =\frac{1}{T}{\int }_{0}^{T}{E}_{pl}^{\mathrm{int}}(t)dt=\frac{1}{N}\sum _{i=1}^{N}{E}_{pl}^{\mathrm{int}}({t}_{i})$$

and

9 $$\langle {e}^{\beta {\rm{\Delta }}{E}_{pl}^{\mathrm{int}}}\rangle =\frac{1}{N}\sum _{i=1}^{N}{e}^{\beta {\rm{\Delta }}{E}_{pl}^{\mathrm{int}}({t}_{i})}$$

where *β* is $$\frac{{\rm{1}}}{KT}$$.

Finally, combine Δ*G*_*sol*_ calculated by the MM/PBSA method with Δ*G*_*gas*_ calculated by the IE method, we can obtain Δ*G*_*blind*_ accurately and efficiently.

### MD simulation

The initial structure of trypsin and two ligands are generated from the Protein Date Bank (entry: 1C5T and 1O2J). The structures of the ligands are optimized at HF/6-31 G** level and the calculation of single point energy is at B3LYP/cc-PVTZ level which is consistent with the Duan *et al*.^46^ to obtain electrostatic potentials (ESP) for fitting their atomic charges using the restrained ESP (RESP)^36^ approach. AMBER12SB force field and the general AMBER force field (GAFF) are used to produce the parameters of the protein and ligands respectively. The truncated periodic octahedral box of TIP3P waters is employed as the solvent environment. There is a buffer of at least 10 Å between the complex and the periodic box wall. The chloride counter ions are added to keep the system electrically neutral. We take advantage of the steepest descent method followed by conjugate gradient minimization to achieve energy minimization and the whole systems with 10 kcal (mol Å^2^)^−1^ restraint are heated from 0 to 300 K continuously for 300 ps with a time step of 2 fs. SHAKE^47^ algorithm is used to constrain all bonds involving hydrogen atoms. Simulations are performed in the NPT ensemble. During the entire process of MD simulations, we run a total of 90 ns. In the first 80 ns, 4 ps per frame is written to the coordinated file. During 80 to 90 ns, the time of per frame is 10 fs to obtain enough conformational sampling.

The MD simulations of PPC force field does not make big change compared with AMBER force field except that the charge of solute is replaced by PPC because of polarization effect. More detailed description about PPC force field has been shown in the part of the Polarized Protein-Specific Charge.

## Results and Discussion

### Analysis of the stability

At first, we compare the results of single system simulations and triple system simulations and find it is not suitable for long-term simulations using triple system simulations. The detailed analysis is shown in the part of the analysis of binding free energy. Therefore, the analysis of the stability is only for single system simulations.

**Figure 1:**
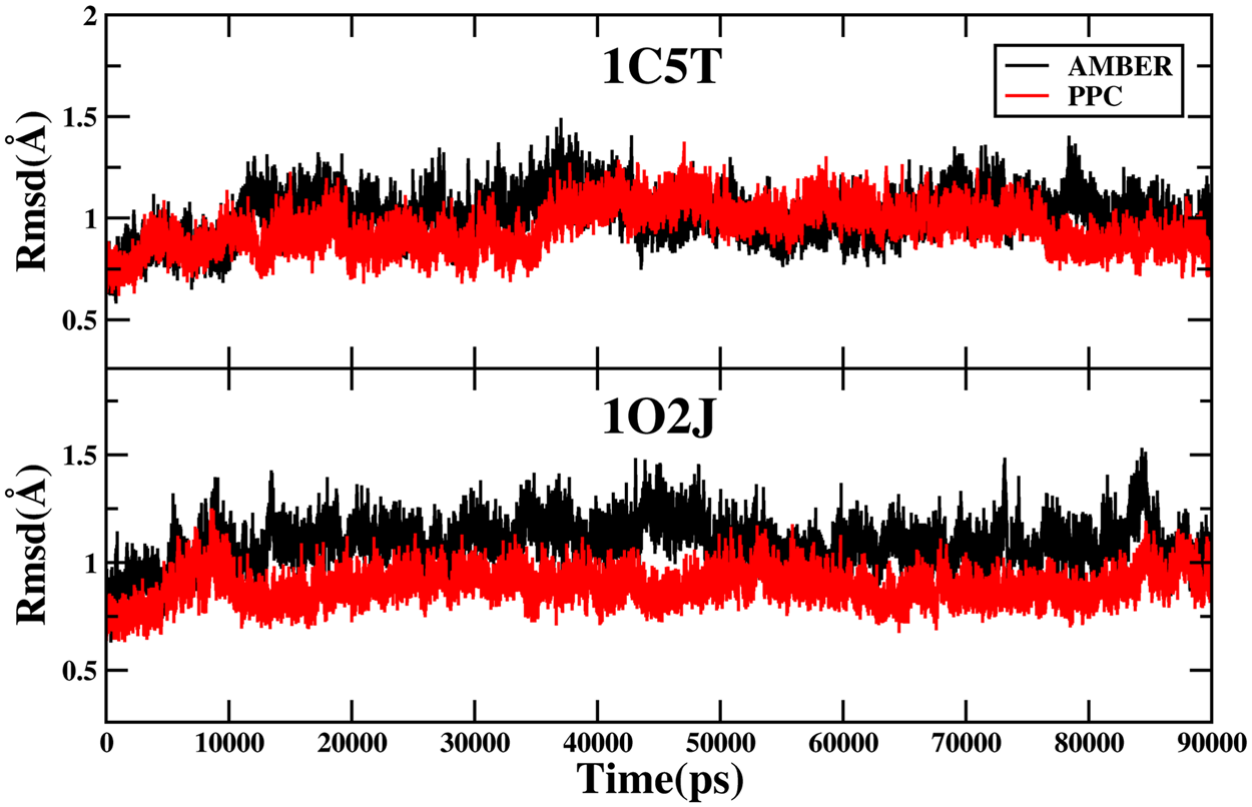
The root-mean-square deviation (RMSD) of the backbone atoms relative to the corresponding native structure during the 90 ns MD simulations. The upper part of the figure is 1C5T system in AMBER (black) and PPC (red); and the lower part of the figure is 1O2J system.

In order to appraise the stability of MD simulations equilibrium, the root mean square deviation (RMSD) of the backbone atoms relative to the corresponding native structure as function of time is calculated and shown in the Fig. 1. As is shown in the figure, most of the values of RMSD are fluctuated between 0.7 Å and 1.4 Å, and MD simulations of the two systems have reached equilibrium after around 15 ns under AMBER and PPC force field, respectively. In addition, there are some differences between AMBER and PPC. For the 1C5T system, the average values of RMSD in AMBER and PPC are 1.01 Å and 0.95 Å, respectively. For the 1O2J system, the average values of RMSD in AMBER and PPC are 1.09 Å and 0.89 Å, respectively. The phenomenon that the average values of RMSD in PPC are smaller than AMBER indicates PPC can make the whole MD simulations more stable. The analysis of the RMSD, on the one hand, suggests that the two systems have reached equilibrium under two simulations, on the other hand, shows that PPC force field can provide more stable MD simulations than AMBER force field.

**Figure 2:**
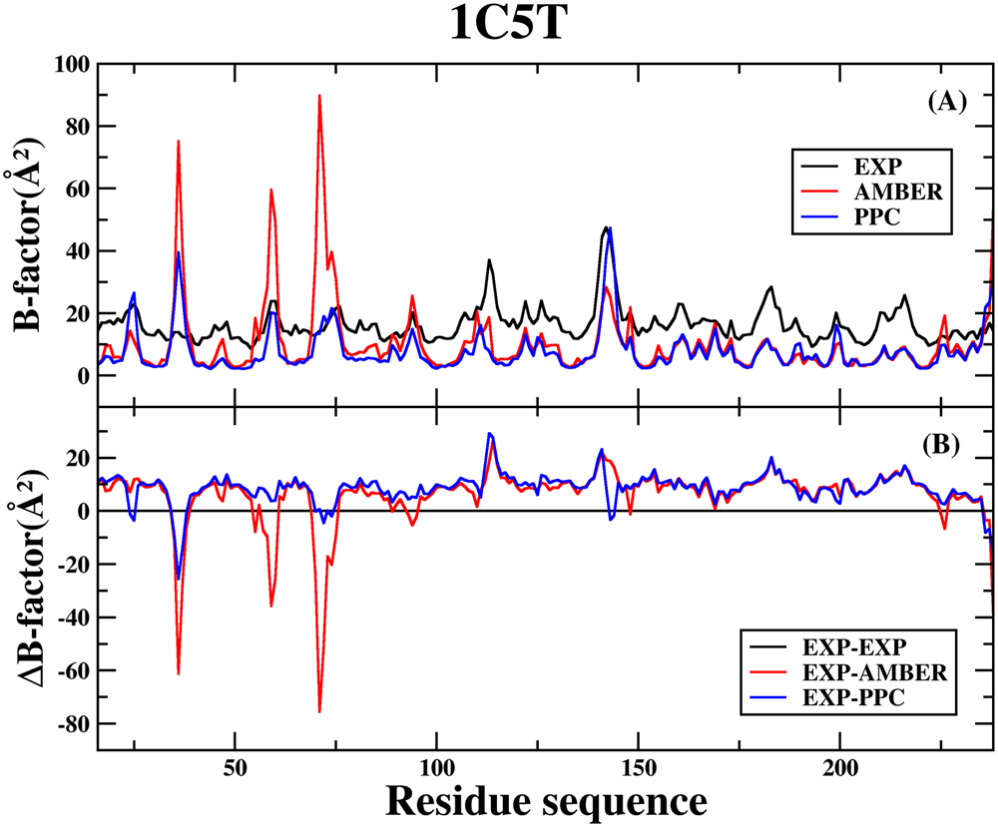
(**A**) The B-factor of protein *C*_*α*_ atoms of the 1C5T system in AMBER, PPC and experiment during 80 to 90 ns MD simulations. (**B**) The difference of B-factor in AMBER, PPC and experiment.

**Figure 3:**
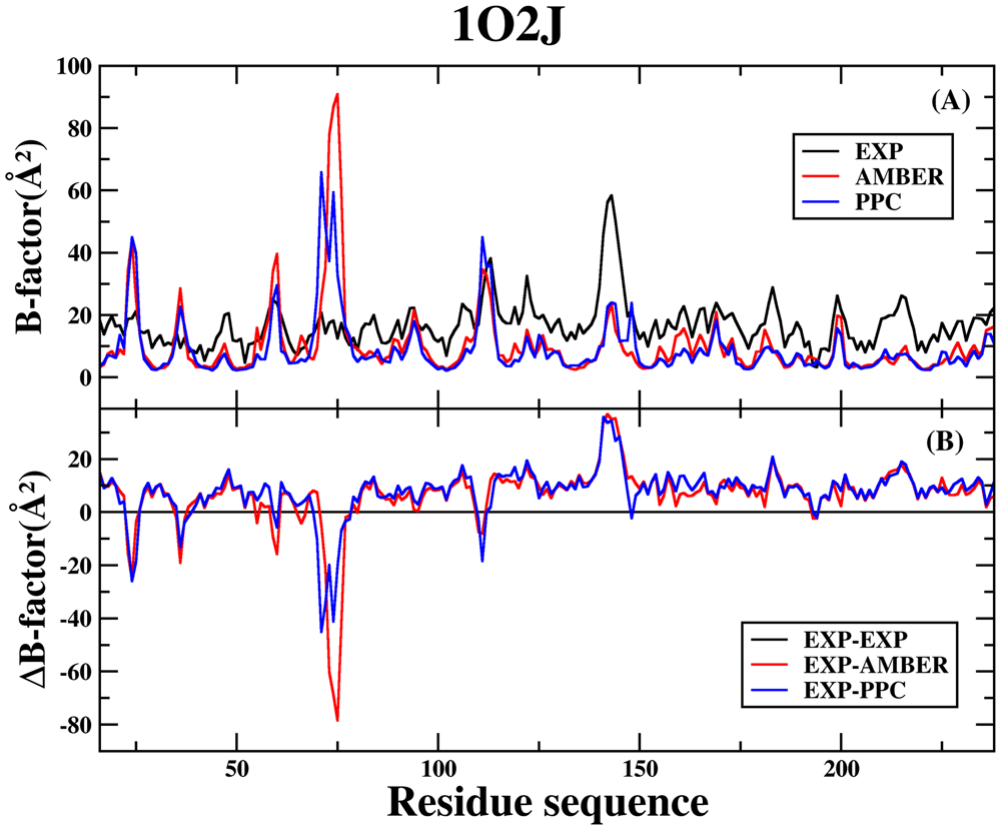
(**A**) The B-factor of protein *C*_*α*_ atoms of the 1O2J system in AMBER, PPC and experiment during 80 to 90 ns MD simulations. **(B)** The difference of B-factor in AMBER, PPC and experiment.

In order to further analyze residual atomic flexibility, an isotropic temperature factor (B-factor) has been calculated under AMBER and PPC. B-factor reflects the mobility of each residue around its mean position, which is another instrument for analyzing the dynamics stability during the simulations. Figures 2 and 3 show the B-factor of protein *C*_*α*_ atoms of the 1C5T and 1O2J systems, respectively.

**Figure 4:**
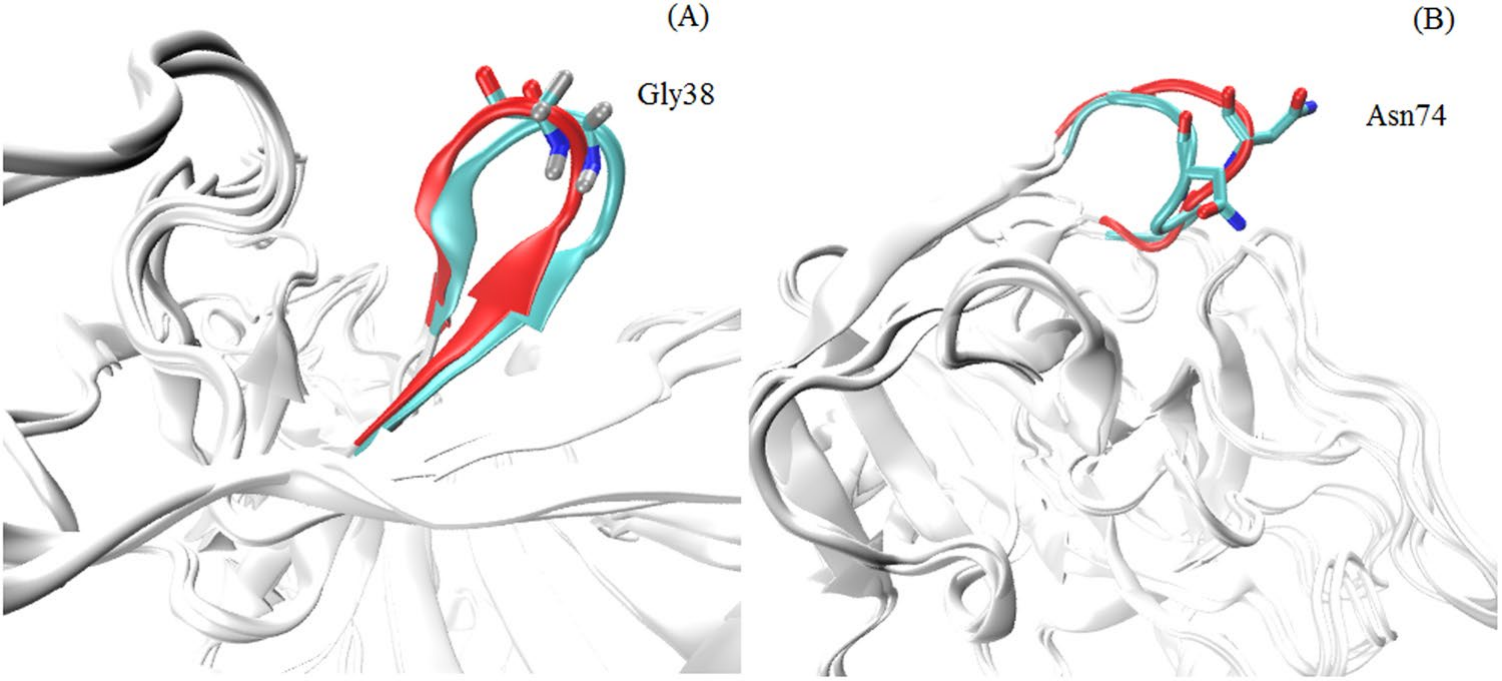
The overlap of the native structure and the simulated lowest potential energy structure under PPC. (**A**) 1C5T system (**B**) 1O2J system.

For the 1C5T system, Fig. 2(A) shows the B-factor of protein *C*_*α*_ atoms in AMBER, PPC and experiment. Figure 2(B) shows the difference of B-factor in AMBER, PPC and experiment. From the general trend of change point of view: the B-factor of AMBER force field and PPC force field is nearly similar with experimental value. However, there are several residues (Gly38, Ser61 and Asn74) with higher flexibility in AMBER than experiment. The reason we analyze may be that the polarization effect is not taken into account in AMBER. This shows the structure from AMBER force field is more unstable than from PPC force field. In addition, there is a strange phenomenon that the area that closes to residue Gly38 shows greater flexibility in PPC than experiment. We analyze the second structure of protein, then find the residue Gly38 is turn structure. The overlap of native structure and the simulated lowest potential energy structure has been shown in the Fig. 4(A). It can obviously notice that the structure of Gly38 does change a lot. It is the unstable structure that leads the B-factor is such large.

For the 1O2J system, Fig. 3(A) shows the B-factor of protein *C*_*α*_ atoms in AMBER, PPC and experiment. Figure 3(B) shows the difference of B-factor in AMBER, PPC and experiment. The result is basically same with above 1C5T system. There are several residues (Val76, Glu77 and Gly78) with higher flexibility in AMBER than experiment. This shows the structure in PPC is more stable than AMBER. The area that closes to residues Asn74 shows greater flexibility in PPC than experiment. The second structure of Asn74 is turn structure, as well. The overlap of native structure and the simulated lowest potential energy structure has been shown in the Fig. 4(B). The unstable structure of Asn74 leads the B-factor is more flexible in PPC force field than experimental observed.

In short, the analysis of B-factor indicates structure of the protein is more stable under the PPC force field, compared to the AMBER force field.

### The analysis of binding free energy

Binding free energy of single system simulations and triple system simulations during 2 to 4 ns have been shown on the Table 1. It can be find that no matter AMBER force field or PPC force field, results of triple system simulations are indeed closer to experimental values. In order to further explore the results of long-term simulations, binding free energy and standard deviations of single system simulations and triple system simulations during 80 to 90 ns are calculated. For single system simulations, the standard deviations are from calculation of 100 snapshots. For triple system simulations, considering that complex, protein and ligand are in motion separately, we take 20 different 100 snapshots structure and calculate 20 groups binding free energy with the same method as before. Binding free energy is the average of 20 results, and the standard deviations are from calculation of 20 results. Detailed results have been shown on Table S1~S4 in the supporting information. Final binding free energy and standard deviations are shown in Table 2 under AMBER force field and Table 3 under PPC force field. Obviously, results of triple system simulations deviate from the experimental value. However, single system simulations are still stable. According to above results, the method of triple system simulations for the two systems may be effective in a short period of time. However, it is not suitable for long-term simulations due to the large conformational change during MD simulations. Therefore, single system simulation is used for further analysis.

**Table 1:** Binding free energy of single system simulations and triple system simulations during 2 to 4 ns.

	1C5T				1O2J			
	AMBER		PPC		AMBER		PPC	
	Single	Triple	Single	Triple	Single	Triple	Single	Triple
Δ*E*_*ele*_	−20.70	−51.83	−28.06	−15.35	−8.87	−29.54	−33.40	42.85
Δ*E*_vdw_	−22.97	−13.95	−20.63	−33.65	−31.61	−48.07	−32.05	−31.11
Δ*E*_int_	0.00	−11.28	0.00	−14.10	0.00	2.64	0.00	−11.69
Δ*G*_*sol*_	6.30	55.27	8.09	18.48	1.93	40.34	22.12	−36.51
−*T*Δ*S*	15.62	11.17	16.41	22.07	20.78	27.69	21.59	18.14
Δ*G*_*blind*_	−21.75	−10.62	−24.19	−22.55	−17.77	−6.94	−21.74	−18.32
${\rm{\Delta }}{G}_{\exp }^{\ast }$	−5.6				−7.8			

The Nmode method is used to compute the entropy contribution. All values are in kcal/mol. *The experimental value is calculated by Caterina Barillari *et al*.^49^.

**Table 2:** Binding free energy and standard deviations in the single system simulations and triple system simulations under AMBER force file.

	1C5T				1O2J			
	Single		Triple		Single		Triple	
	MEAN	STD	MEAN	STD	MEAN	STD	MEAN	STD
ΔE_ele_	−22.36	6.94	−35.74	4.22	−15.22	8.56	127.65	4.65
ΔE_vdw_	−21.83	3.03	−20.63	2.85	−31.82	3.65	−22.19	2.44
ΔE_int_	0.00	0.00	−17.57	3.88	0.00	0.00	−7.51	6.95
ΔG_sol_	8.34	6.09	14.27	2.97	10.61	8.69	−166.82	4.16
−TΔS	19.58	2.12	16.04	1.69	18.20	6.16	18.77	1.44
ΔG_blind_	−16.27		−43.63		−18.23		−50.10	
$\Delta {G}_{\exp }^{\ast }$	−5.6				−7.8			

The Nmode method is used to compute the entropy contribution. All values are in kcal/mol.

**Table 3:** Binding free energy and standard deviations in the single system simulations and triple system simulations under PPC force file.

	1C5T				1O2J			
	Single		Triple		Single		Triple	
	MEAN	STD	MEAN	STD	MEAN	STD	MEAN	STD
ΔE_ele_	−32.05	8.55	79.59	8.89	−27.24	7.99	−58.16	8.04
ΔE_vdw_	−21.00	3.27	−43.66	4.15	−28.32	2.86	−16.21	3.68
ΔE_int_	0.00	0.00	−13.28	9.69	0.00	0.00	−19.55	5.35
ΔG_sol_	15.18	6.72	−42.09	4.31	15.76	7.03	25.25	6.05
−TΔS	17.28	2.68	21.58	6.03	20.73	4.69	22.17	4.30
ΔG_blind_	−20.59		2.14		−19.07		−46.50	
${\rm{\Delta }}{G}_{\exp }^{\ast }$	−5.6				−7.8			

The Nmode method is used to compute the entropy contribution. All values are in kcal/mol.

**Figure 5:**
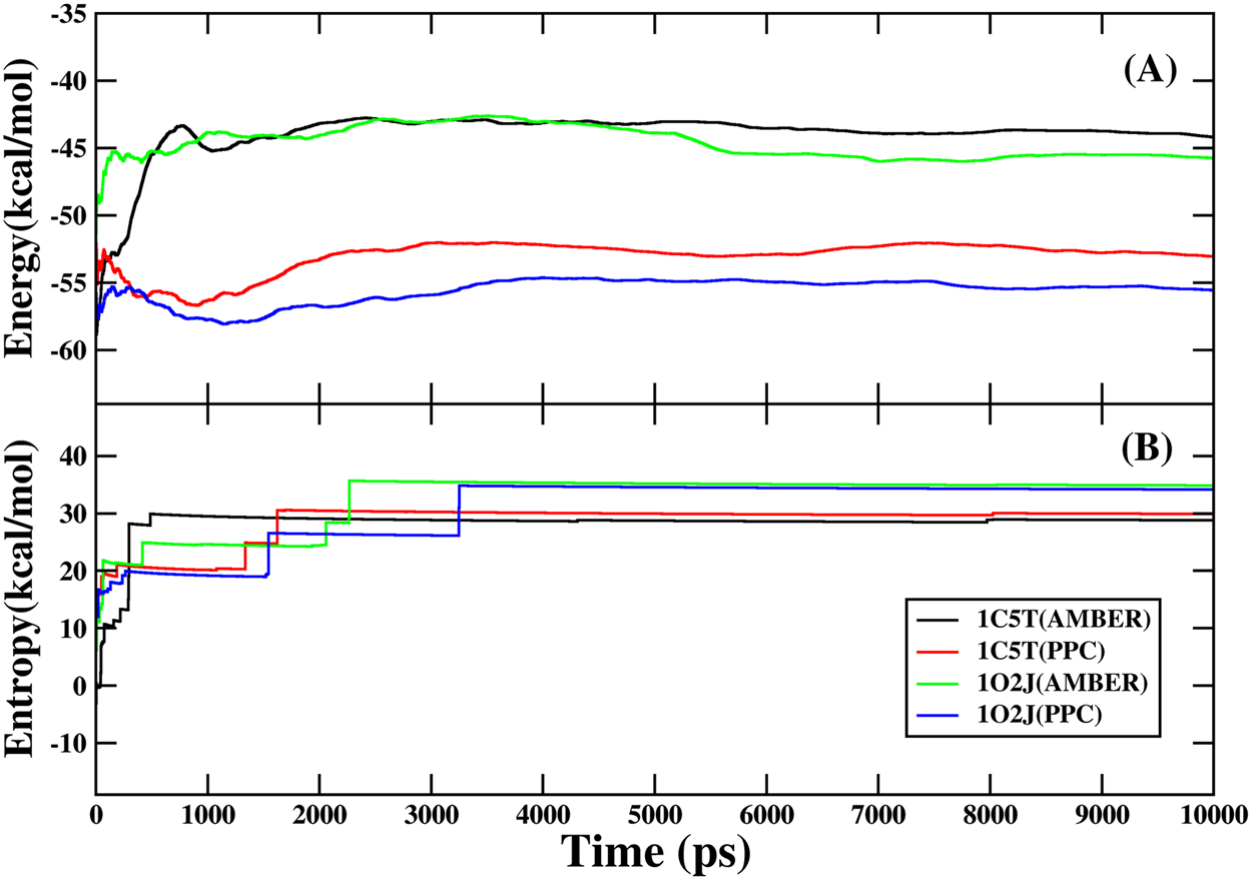
(**A**) Interaction energy between trypsin and ligand in AMBER and PPC during 80 to 90 ns of MD simulations. (**B**) Interaction entropy between trypsin and ligand in AMBER and PPC during 80 to 90 ns of MD simulations.

We should ensure that the average interaction energy and interaction entropy calculated by IE method have reached convergence, before analyzing the binding free energy of protein and ligand. Figure 5 shows the fluctuation of average interaction energy $(\langle {E}_{pl}^{\mathrm{int}}\rangle )$ and interaction entropy (−*T*Δ*S*) in the mean over time during 80 to 90 ns of MD simulations. It suggests that the interaction energy and interaction entropy of two systems is wonderfully converged with respect to abundant conformation sampling. What’s more, it is noticed obviously that interaction energy in PPC is lower than AMBER from Fig. 5(A). Figure 5(B) shows that the interaction entropy obtained from AMBER and PPC is nearly the same whether for 1C5T or 1O2J. The results may indicate that PPC which takes the polarization into account mainly affects the interaction energy, and doesn’t make significant differences on interaction entropy, compared with AMBER force field.

In order to investigate the polarization effect and effectiveness of the IE method on the trypsin protein, in our works, two MD simulations (AMBER and PPC force field) and two methods (Nmode and IE method) are combined to calculate the binding free energy between trypsin and ligand for the two systems: (1) the MD simulation using AMBER force field and the calculation of entropy change using Nmode method, (2) the MD simulation using AMBER force field and the calculation of entropy change using IE method, (3) the MD simulation using PPC force field and the calculation of entropy change using Nmode method, (4) the MD simulation using PPC force field and the calculation of entropy change using IE method. Those results have been displayed in the Table 4. In the calculation of binding free energy, the total energy is divided into three sections: the protein-ligand interaction energy $(\langle {E}_{pl}^{\mathrm{int}}\rangle )$, the solvation free energy (Δ*G*_*sol*_) and the entropy contribution (−*T*Δ*S*). Considering that we employ the same modus to calculate $\langle {E}_{pl}^{\mathrm{int}}\rangle$ and Δ*G*_*sol*_ parts of the binding free energy in the method of Nmode and IE, the only divergence in calculation is −*T*Δ*S* parts of the binding free energy under the same force field. As is shown in the Table 4, the difference of −*T*Δ*S* between Nmode and IE plays a significant role in calculation of binding free energy. In addition, comparing AMBER with PPC, we can discover obviously that the key discrepancy is mainly reflected in $\langle {E}_{pl}^{\mathrm{int}}\rangle$ and Δ*G*_*sol*_ two components. The contributions of −*T*Δ*S* toward binding free energy are basically identical between AMBER and PPC under the same method for the calculation of entropy change. This result is consistent with the previous analysis of entropy contribution^48^ that they are nearly the same under two force field shown in Fig. 5.

**Table 4:** Binding free energy between trypsin and ligand in AMBER and PPC force field during 80 to 90 ns MD simulations.

PDB code	Method	${\boldsymbol{\langle }}{{\boldsymbol{E}}}_{{\boldsymbol{p}}{\boldsymbol{l}}}^{{\bf{int}}}{\boldsymbol{\rangle }}$	Δ*G*_*sol*_	−*T*Δ*S*		Δ*G*_*blind*_		${\rm{\Delta }}{G}_{\exp }^{\ast }$
				*N*_mode_	IE	*N*_mode_	IE	
1C5T	AMBER	−44.19	8.34	19.58	27.62	−16.27	−8.23	−5.6
	PPC	−53.05	15.18	17.28	29.99	−20.59	−7.88	
1O2J	AMBER	−47.04	10.61	18.20	34.86	−18.23	−1.57	−7.8
	PPC	−55.56	15.76	20.73	34.15	−19.07	−5.65	

The Nmode and IE methods are used to compute the entropy contribution, respectively .All values are in kcal/mol.

**Figure 6:**
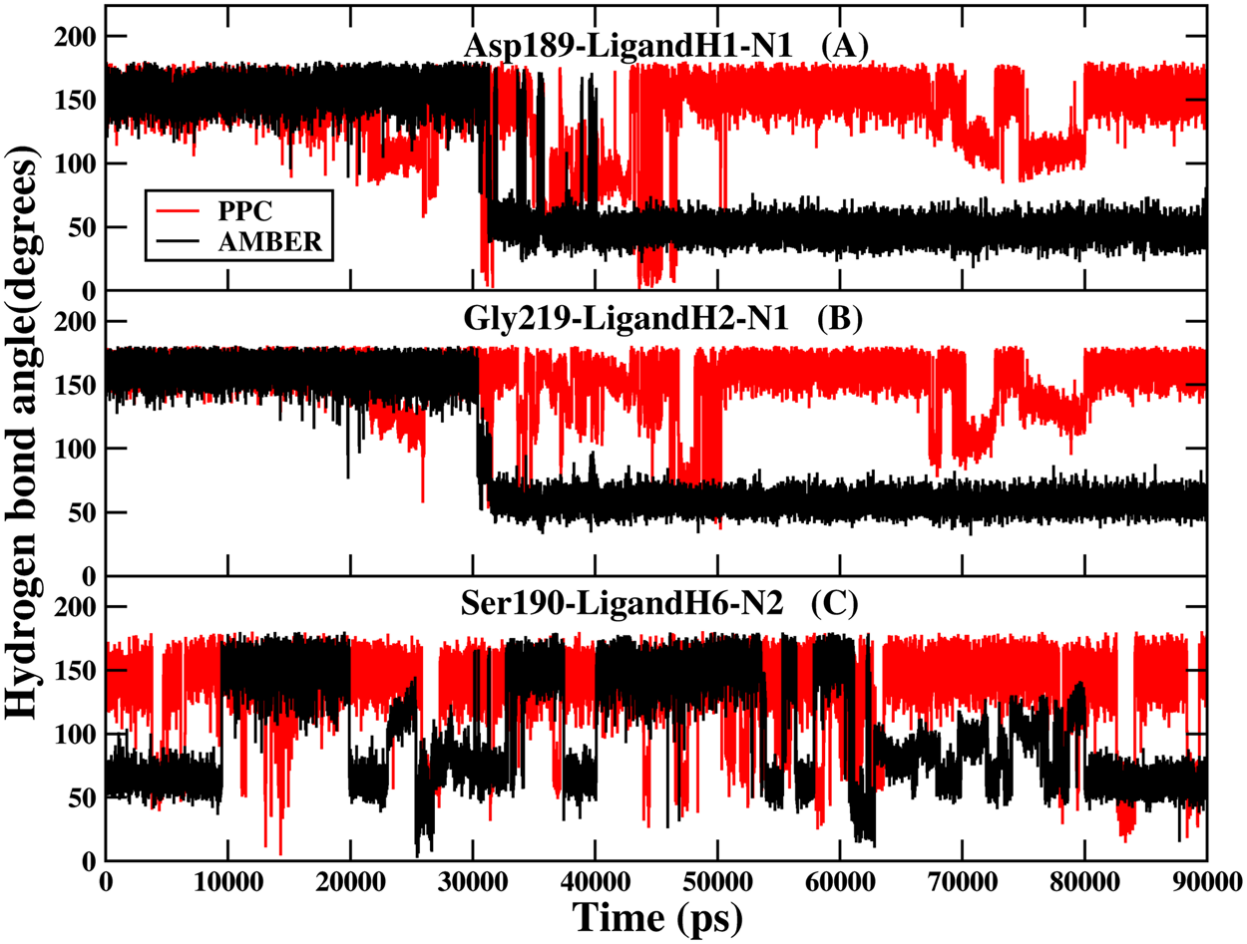
The angles of three hydrogen bonds under AMBER (black) and PPC (red) as the function of MD simulations. (**A**) and (**B**) are for 1C5T system. (**C**) Is for 1O2J system.

The reason for the discrepancy of term $\langle {E}_{pl}^{\mathrm{int}}\rangle$ in AMBER and PPC comes from the broken hydrogen bonds between trypsin and ligand in MD simulation using AMBER force field. The distance and angle of hydrogen bond formed between trypsin and ligand in the two systems under AMBER and PPC force field are further analyzed. Although the distances of those hydrogen bonds have no obvious difference which are fluctuated around 3.5 Å under the two force fields, their angles are broken during the MD simulation using AMBER force field. The Fig. 6(A) and (B) show the angles of hydrogen bonds formed between Asp189-LigandH1-N1 and Gly219-LigandH2-N1 under AMBER and PPC in 1C5T system. And Fig. 6(C) shows the angle of hydrogen bond formed between Ser190-LigandH6-N2 under AMBER and PPC in 1O2J system, respectively. It can be seen obviously that hydrogen bonds Asp189-LigandH1-N1 and Gly219-LigandH2-N1 are broken after approximately 30 ns under AMBER, and hydrogen bond Ser190-LigandH6-N2 is broken completely after approximately 63 ns under AMBER. However, these three hydrogen bonds are very stable under PPC force field. Due to the stable hydrogen bond formed between trypsin and ligand using PPC force field, the calculated interaction energy is stronger using PPC force field than AMBER force field.

From Table 4, the result of PPC force field combined with the IE method is in excellent agreement with experimental value compared with the results calculated by other three combinations. The experimental values of 1C5T and 1O2J are −5.6 kcal/mol and −7.8 kcal/mol, respectively. And the calculated value of 1C5T and 1O2J in the method of PPC and IE are −7.88 kcal/mol and −5.65 kcal/mol, respectively. The gap between experimental values and calculated value is approximate 2 kcal/mol. This result indicates that the electrostatic polarization plays a significant role in MD simulation and the IE method is superior to the Nmode method in binding free energy prediction. The combination of PPC force field and IE method is the best choice of the four methods in MD simulation and the calculation of binding free energy.

Of course, above result only analyzes 10 ns segment of MD trajectories. It may be accidental and can’t prove the combination of PPC force field and IE method has a general superiority. Therefore we run continuously 200 ns MD trajectories on the basis of the original and take 20 additional 10 ns segments and perform the same analysis. The RMSD of 200 ns MD simulation is shown on Fig. S1 in the supporting information, and it is basically the same as the original result. The detailed free energies of 1C5T and 1O2J are shown on Tables S5 and S6 in the supporting information, respectively. For 1C5T, the four average values of 20 sets of results are −18.79 kcal/mol, −8.10 kcal/mol, −21.91 kcal/mol, −7.17 kcal/mol and the standard deviations (STD) are 1.42 kcal/mol, 0.93 kcal/mol, 1.44 kcal/mol and 0.52 kcal/mol. For 1O2J, the four average values of 20 sets of results are −13.11 kcal/mol, −0.35 kcal/mol, −17.44 kcal/mol, −4.72 kcal/mol and the STD are 2.38 kcal/mol, 2.35 kcal/mol, 1.42 kcal/mol and 1.51 kcal/mol. Besides, the distributions of results have been plotted on Figs S2 and S3 in the supporting information. Although there is difference between 1C5T and 1O2J, results of these 20 groups are basically distributed around the original results, without significant fluctuations. This can indicate that the results of the two system are statistically persuasive.

### The decomposition of residue

**Figure 7:**
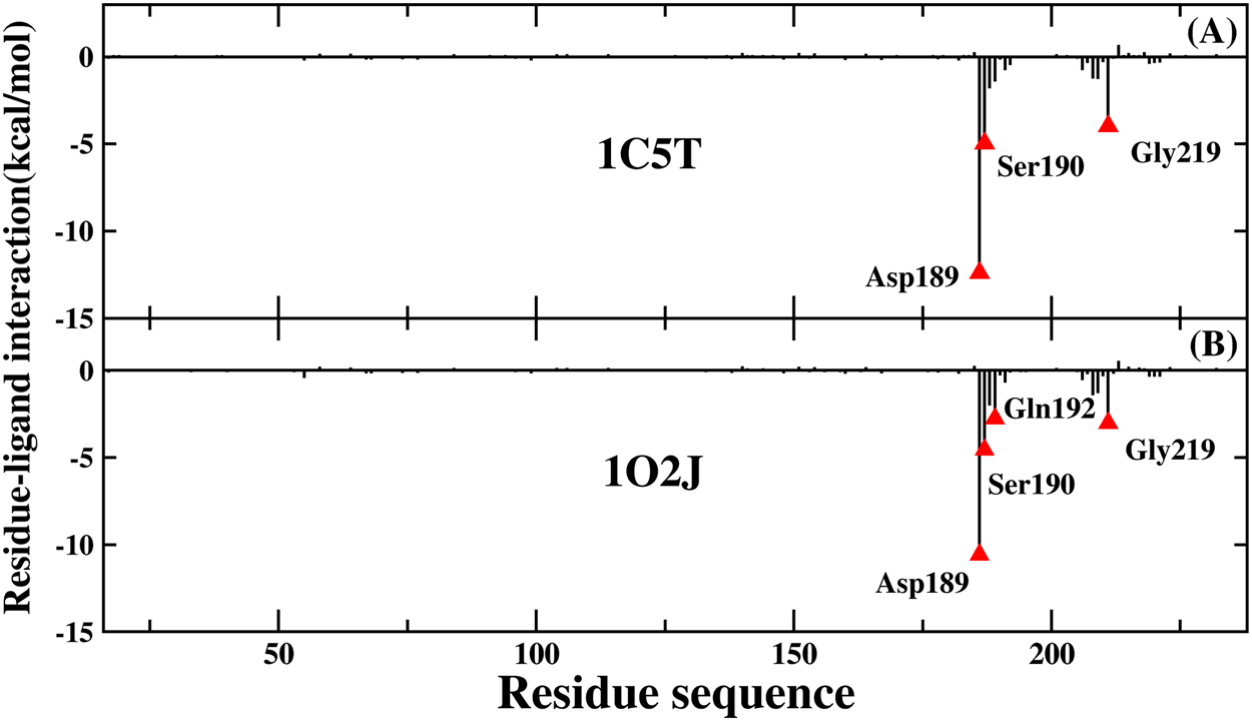
Decomposition of the binding free energy on a per-residue basis for trypsin-ligands complexes in two systems based on PPC.

**Figure 8:**
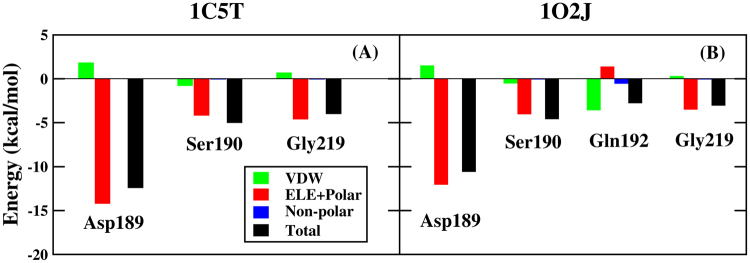
Decomposition of the binding free energy on a per-basis into contributions from vdW energy, the sum of electrostatic energy and polar solvation energy, non-polar solvation energy based on PPC.

In order to further explore the binding mechanism of protein-ligand, the contribution of every individual residue toward the binding free energy has been analyzed in detail. The binding free energy is decomposed into residue-ligand pairs to generate residue-ligand interaction spectrum, shown in Fig. 7. The means of the residue decomposition is extremely helpful to explain the binding mechanism of protein-ligand at atomic level and beneficial to analyze the contribution of each individual residue to the binding free energy, as well. According to the analytic result of residue-ligand interaction spectrum, the contribution toward binding free energy of several key residue-ligand pairs is decomposed into vdW energy, the sum of electrostatic energy and polar solvation energy, and non-polar solvation energy. The result has been shown in Fig. 8. The advantage of PPC over traditional AMBER force field has been shown from the above analysis, and the decomposition of residue is only performed in PPC force field.

Figure 7(A) illustrates the residue-ligand interaction spectra of 1C5T system. The major contribution toward binding free energy comes from a few groups around Asp189-ligand, Ser190-ligand and Gly219-ligand. Among them, the complex of Asp189-ligand plays an extremely prominent role on the contribution toward binding free energy. And the following analysis of hydrogen bond shows that there is strong hydrogen bond interaction between residue Asp189 and ligand. Figure 8(A) illustrates the decomposition of energy of several pivotal residue-ligand pairs in 1C5T system. It is obvious that the sum of electrostatic energy and polar solvation energy plays a significant part in contribution toward binding free energy.

Figure 7(B) illustrates the residue-ligand interaction spectra for 1O2J system. The major contribution toward binding free energy comes from a few groups around Asp189-ligand, Ser190-ligand, Gln192-ligand and Gly219-ligand. Among them, the complex of Asp189-ligand also makes extremely significant contribution towards binding free energy. Figure 8(B) illustrates the decomposition of energy of several pivotal residue-ligand pairs in 1O2J. For Asp189-ligand, Ser190-ligand and Gly219-ligand, the sum of electrostatic energy and polar solvation energy plays a significant part in contribution toward binding free energy, as well. However, vdW interaction plays an important role during the binding of Gln192 and ligand with the value of −3.56 kcal/mol. The point can be explained by the following analysis of hydrogen bond.

### The analysis of hydrogen bond

As a link between protein and ligand, the hydrogen bond is extremely considerable when analyzing the interaction between protein and ligand. In addition, by the above decomposition of residue, we speculate there may be strong hydrogen bond interaction between residue Asp189 and ligand in the systems of 1C5T and 1O2J. Therefore, in order to verify our judgment, in the next step, the analysis of hydrogen bond between protein and ligand is performed under PPC force field. As is shown in the Table 5, several main hydrogen bonds between protein and ligand are listed during 80 to 90 ns MD simulation. In 1C5T system, there are three stable hydrogen bonds formed between residue Asp189 and ligand with the high occupancy of 99.85%, 99.82% and 96.15% under PPC force field. In 1O2J system, Asp189 is linked by two stable hydrogen bonds with ligand with the occupancy of 99.99% and 76.88%. This explains excellently the strong interaction between Asp189 and ligand observed in the decomposition of residue. Besides, Ser190 and Gly219 also form stable hydrogen bonds with ligand with high occupancy in the two systems from Table 5. However, in 1O2J system, no hydrogen bond forms between residue Gln192 and ligand, though Gln192 have a significant contribution toward the binding free energy based on the analysis of decomposition of residue. The decomposition of energy in Fig. 8(B) has found that vdW interaction plays an important role during the binding of Gln192 and ligand. This result is consistent with the previous decomposition of residue.

**Table 5:** Occupancy of hydrogen bonds between trypsin and ligand during 80 to 90 ns MD simulations based in PPC force field.

PDB code	Acceptor	Donor	Distance (Å)	Angle (°)	Occupancy (%)
1C5T	Gly219O	LigandN1-H2	2.7891	162.7407	100.00
	Asp189OD1	LigandN1-H1	2.7533	155.0623	99.85
	Asp189OD2	LigandN2-H3	2.7953	152.1022	99.82
	Ser190OG	LigandN2-H4	2.9636	150.2597	98.76
	Asp189OD1	LigandN2-H3	3.0460	137.2535	96.15
1O2J	Asp189OD1	LigandN1-H5	2.7542	161.3021	99.99
	Gly219O	LigandN1-H4	2.8339	157.5889	99.94
	Asp189OD2	LigandN2-H7	2.7553	155.0558	76.88
	Ser190OG	LigandN2-H6	2.9507	150.3367	73.57

**Figure 9:**
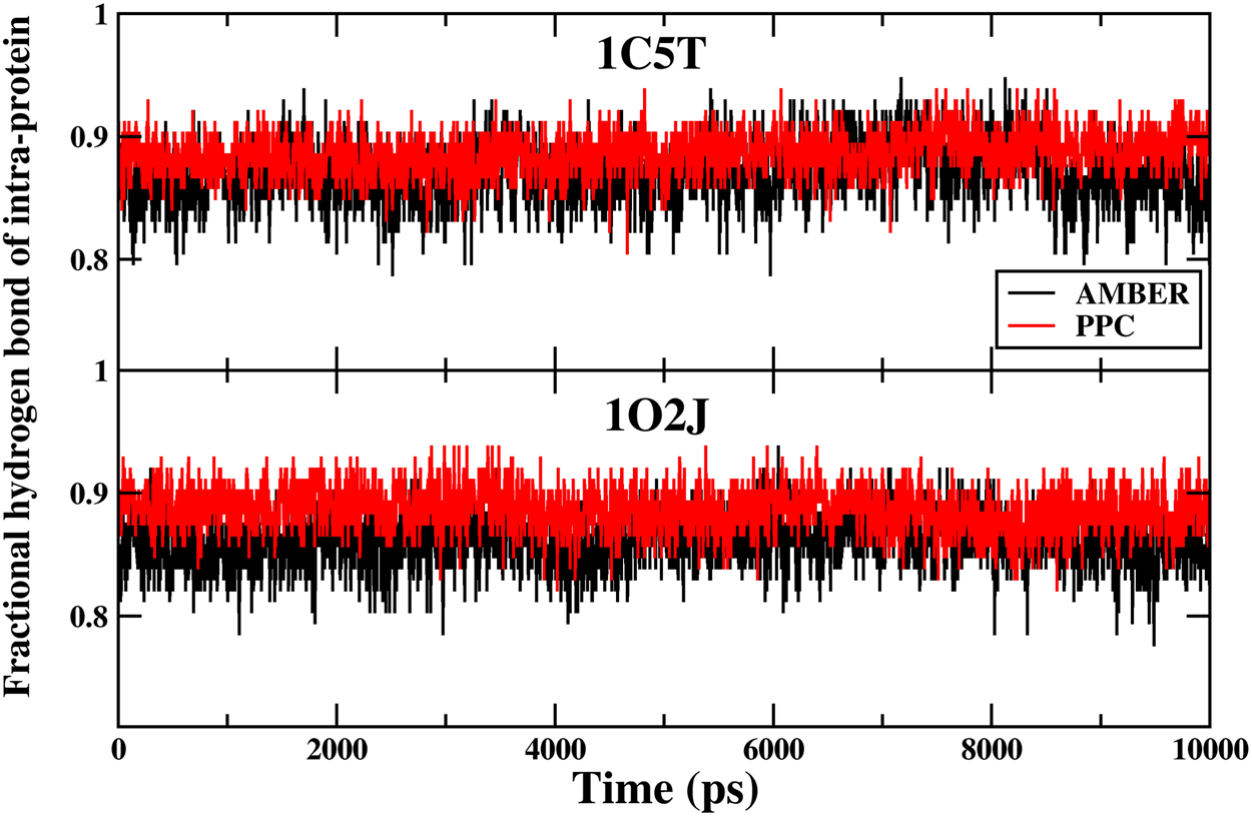
The time evolution of the fractional native hydrogen bonds in intra-protein during 80 to 90 ns under AMBER (black) and PPC (red).

Hydrogen bond is important not only for the binding of proteins and ligands, but also for the stability of the internal structure of the protein. So, the number of hydrogen bonds in intra-protein is further analyzed. The hydrogen bonds should be more stable in PPC than in AMBER during the MD simulation, because electrostatic polarization can affect the interaction energies of hydrogen bonds. At first, we detect the number of hydrogen bonds in intra-protein in the native structure. The hydrogen bond length and angle cutoffs are 3.5 Å and 120°, respectively. There are a total of 113 and 112 hydrogen bonds in the systems of 1C5T and 1O2J, respectively. Then the time evolution of the fractional native hydrogen bonds in intra-protein during 80 to 90 ns is shown in the Fig. 9. Fractional number of hydrogen bonds is the number of hydrogen bonds in intra-protein presented in the simulation structures divided by the total in the native structure. From the figure, we can find that the fractional number of hydrogen bonds is higher in PPC than AMBER. The average fractional number of 1C5T in AMBER and PPC is 0.87 and 0.98, respectively. And the average fractional number of 1O2J system in AMBER and PPC is 0.86 and 0.89, respectively. The result suggests that more hydrogen bonds are preserved in PPC than in AMBER, which is consistent with previous finding.

## Conclusions

The current study emphasizes the MD simulation under the polarizable force field PPC combined with IE method is precise and efficient for studying the mechanism of the interaction between trypsin and ligand. Especially, the results that we calculate can provide useful information for drug design. In our report, single system and triple system simulations are performed to calculate binding free energy. In a short period of time, triple system simulations are closer to experimental values. However, for long-term simulations, results of triple system simulations deviate from the experimental value. Therefore, our analysis is mainly based on single system simulations.

In single system simulations, four methods are employed to calculate the binding free energy of trypsin and ligands: (1) AMBER force field combined with Nmode, (2) AMBER force field combined with IE, (3) PPC force field combined with Nmode, (4) PPC force field combined with IE. According to the analyses of the RMSD, B-factor, intra-protein and protein-ligand hydrogen bonds, we find the structure of the protein is more stable under the PPC force field, comparing to the AMBER force field. As far as the binding free energy is concerned, our study discovery the binding free energy of 1C5T and 1O2J system under PPC forced field combined with IE method is in excellent agreement with experimental value. Here we analyze the reasons for the four different results. For AMBER and PPC, the only difference is that AMBER force field does not take the effect of electrostatic polarization into consideration. For the two methods of Nmode and IE for calculating entropy change, IE calculates the entropy change through a more efficient and rigorous formula, and has abundant ensemble sampling. During the calculation of the interaction entropy, all snapshots are exacted from the MD trajectories in the IE method while the Nmode method only selects 10 snapshots which may lead to some errors in the calculation results. Of course, any theoretical value can’t be exactly the same as the experimental value, the possible sources of error we analyze are listed. (1) The simulation environment is not exactly the same as the experimental environment. (2) The accuracy of the force field during the MD simulation. (3) The calculation of the solvation free energy is based on the implicit solvent mode while MD simulation is based on the explicit solvent mode.

Furthermore, to further understand interaction mechanisms between proteins and ligands, the detailed analysis of residue decomposition are carried out under PPC force field. The results show the Asp189, Ser190, Gln192 and Gly219 play a dominant favorable role in the binding free energy. For this phenomenon, we decompose the binding free energy into vdW energy, the sum of electrostatic energy and polar solvation energy, and non-polar solvation energy based on the main residues, finding the sum of electrostatic energy and polar solvation energy play a vital role in the binding free energy. Further analysis finds the hydrogen bonds between Asp189, Ser190, Gln219 and ligand are quite stable and the vdW energy of Gln192 and ligand is extremely strong. Therefore, these residues make a significant contribution to maintain the binding between the ligand and protein. The information obtained from the current studies provides important insights on the trypsin-ligand binding mechanism and it will be useful for the new drug design in the further.

## Electronic supplementary material

supporting information
