# Supplementary material for: Trypsin-Ligand binding affinities calculated using an effective interaction entropy method under polarized force field
Source: Sci Rep. 2017 Dec 18;7:17708. doi: 10.1038/s41598-017-17868-z (PMC5735144; doi:10.1038/s41598-017-17868-z)

Trypsin-Ligand binding affinities calculated using an effective interaction entropy method under polarized force field

Yalong Cong,[§,1] Mengxin Li, [§,1] Guoqiang Feng,[1] Yuchen Li,[1] Xianwei Wang,[2] Lili. Duan,[*,1]

[1]Shandong Province Key Laboratory of Medical Physics and Image Processing Technology, School of Physics and Electronics, Shandong Normal University, Jinan 250014, China

[2]Center for Optics & Optoelectronics Research, College of Science, Zhejiang University of Technology, Hangzhou 310023, China

Corresponding author (duanll@sdnu.edu.cn;)

[§]Yalong Cong and Mengxin Li contributed equally to this work

Table S1 20 groups detailed binding free energy of triple system simulations in AMBER force field during 80 to 90 ns MD simulation for the 1C5T system. All values are in kcal/mol.

	ΔE_{ele}	ΔE_{vdw}	ΔE_{int}	ΔG_{sol}	$-T\Delta S$	ΔG_{bind}
1	-40.90	-17.68	-17.99	12.73	14.65	-49.19
2	-36.35	-25.62	-19.79	13.98	11.68	-56.1
3	-38.42	-20.39	-20.61	15.98	14.91	-48.53
4	-32.91	-15.22	-21.32	10.94	15.86	-42.65
5	-37.23	-21.87	-13.65	16.06	15.23	-41.46
6	-38.50	-17.41	-15.47	12.88	17.57	-40.93
7	-29.01	-17.40	-19.80	10.70	14.81	-40.7
8	-41.13	-18.18	-19.22	17.16	13.90	-47.47
9	-31.80	-22.43	-12.81	13.77	17.86	-35.41
10	-42.46	-20.84	-12.71	21.40	13.97	-40.64
11	-34.62	-24.71	-19.50	17.45	15.50	-45.88
12	-37.84	-22.26	-17.53	17.41	17.29	-42.93
13	-35.89	-17.58	-15.25	16.13	17.27	-35.32
14	-37.52	-23.77	-23.69	17.07	17.30	-50.61
15	-25.85	-24.69	-21.03	10.89	17.33	-43.35
16	-29.62	-21.39	-23.91	8.45	16.24	-50.23
17	-34.72	-20.32	-8.77	12.61	17.72	-33.48
18	-33.92	-23.09	-19.22	13.30	18.60	-44.33
19	-35.76	-19.07	-15.31	12.79	17.42	-39.93
20	-40.27	-18.64	-13.74	13.75	15.71	-43.19
AVE	-35.74	-20.63	-17.57	14.27	16.04	-43.63
STD	4.22	2.85	3.88	2.97	1.69	5.51

Table S2 20 groups detailed binding free energy of triple system simulations in PPC force field during 80 to 90 ns MD simulation for the 1C5T system. All values are in kcal/mol.

	ΔE_{ele}	ΔE_{vdw}	ΔE_{int}	ΔG_{sol}	$-T\Delta S$	ΔG_{bind}
1	67.97	-39.58	0.26	-38.31	21.69	12.03
2	79.64	-42.6	-29.32	-40.5	32.82	0.04
3	95.25	-49.68	-27.48	-43.72	22.87	-2.76
4	88.22	-49.22	-9.09	-45.85	21.1	5.16
5	89.93	-41.7	-3.02	-48.96	17	13.25
6	93.14	-48.54	-13.06	-49.7	19.5	1.34
7	86.46	-44.98	-6.82	-43.05	16.93	8.54
8	91.39	-43.87	-27.71	-47.02	28.13	0.92
9	89.06	-48.25	-25.8	-50.11	25.79	-9.31
10	74.86	-51.87	-12.7	-35.42	23.74	-1.39
11	67.47	-41.5	-8.32	-37.52	25.33	5.46
12	74.87	-40.93	-16.35	-40.79	16.25	-6.95
13	76.49	-43.91	-8.85	-39.8	19.99	3.92
14	70.29	-41.45	-1.2	-38.05	5.98	-4.43
15	75.16	-36.81	-12.04	-42.7	21.86	5.47
16	79.32	-39.31	-7.16	-39.02	21.07	14.9
17	72.24	-39.81	-19.36	-36.29	24.68	1.46
18	69.6	-44.03	2.4	-40.69	16.8	4.08
19	69.7	-38.08	-13.26	-40.07	16.64	-5.07
20	80.67	-47.13	-26.8	-44.26	33.44	-4.08
AVE	79.59	-43.66	-13.28	-42.09	21.58	2.14
STD	8.89	4.15	9.69	4.31	6.03	6.54

Table S3 20 groups detailed binding free energy of triple system simulations in AMBER force field during 80 to 90 ns MD simulation for the 1O2J system. All values are in kcal/mol.

	ΔE_{ele}	ΔE_{vdw}	ΔE_{int}	ΔG_{sol}	$-T\Delta S$	ΔG_{bind}
1	135.42	-22.79	-11.53	-169.96	17.23	-51.63
2	128.12	-21.63	-16.9	-166.95	20.89	-56.47
3	126.90	-20.32	-10.57	-169.7	19.86	-53.83
4	132.18	-23.78	-3.97	-168.73	18.98	-45.32
5	124.13	-24.2	1.61	-161.24	17.86	-41.84
6	125.13	-24.42	1.95	-158.98	21.57	-34.75
7	129.14	-26.09	5.97	-164.54	19.91	-35.61
8	117.23	-20.58	-24.48	-160.58	20.84	-67.57
9	119.04	-23.37	-10.47	-163.49	19.55	-58.74
10	124.52	-19.67	-5.85	-165.7	19.81	-46.89
11	124.23	-22.63	-12.73	-162.64	18.9	-54.87
12	134.39	-20.09	-7.45	-172.47	16.81	-48.81
13	125.23	-17.43	-9.22	-164.82	18.93	-47.31
14	129.08	-17.01	-13.37	-170.55	17.72	-54.13
15	133.49	-24.03	-6.14	-170.31	17.66	-49.33
16	130.32	-19.77	-8.17	-169.06	19.2	-47.48
17	125.00	-24.47	-0.06	-162.02	18.41	-43.14
18	128.58	-24.56	-0.79	-172.21	16.11	-52.87
19	128.52	-23.11	-11.53	-171.43	18.34	-59.21
20	132.37	-23.82	-6.42	-170.96	16.86	-51.97
AVE	127.65	-22.19	-7.51	-166.82	18.77	-50.10
STD	4.65	2.44	6.95	4.16	1.44	7.69

Table S4 20 groups detailed binding free energy of triple system simulations in PPC force field during 80 to 90 ns MD simulation for the 1O2J system. All values are in kcal/mol.

	ΔE_{ele}	ΔE_{vdw}	ΔE_{int}	ΔG_{sol}	$-T\Delta S$	ΔG_{bind}
1	-81.67	-13.49	-20.65	43.65	22.16	-50.00
2	-64.13	-9.96	-24.06	29.93	25.03	-43.19
3	-47.48	-16.44	-22.75	17.00	22.54	-47.13
4	-63.72	-15.46	-19.65	23.76	21.11	-53.96
5	-60.49	-15.02	-13.40	22.84	15.44	-50.63
6	-44.59	-19.26	-11.70	17.16	26.72	-31.67
7	-50.11	-18.93	-13.43	23.91	15.03	-43.53
8	-54.30	-12.89	-26.70	18.24	22.37	-53.28
9	-52.30	-15.69	-23.71	23.35	25.53	-42.82
10	-61.44	-24.41	-19.96	27.54	25.74	-52.53
11	-61.63	-21.81	-19.98	25.26	32.37	-45.79
12	-57.88	-12.90	-18.50	24.32	20.70	-44.26
13	-61.44	-18.60	-35.24	30.64	21.64	-63.00
14	-50.15	-19.91	-12.37	20.41	20.88	-41.14
15	-54.07	-17.10	-21.70	22.22	23.86	-46.79
16	-58.80	-18.46	-17.81	27.23	21.29	-46.55
17	-53.38	-11.97	-15.48	18.97	25.77	-36.09
18	-64.99	-10.73	-16.27	27.39	19.31	-45.29
19	-65.83	-13.27	-18.65	32.05	23.05	-42.65
20	-54.83	-17.90	-18.99	29.08	12.94	-49.70
AVE	-58.16	-16.21	-19.55	25.25	22.17	-46.50
STD	8.04	3.68	5.35	6.05	4.30	6.57

Table S5 Binding free energy between trypsin and ligand in AMBER and PPC force field during the 20 additional 10 ns MD simulation for the 1C5T system. ΔH represents the total of protein–ligand interaction ($\langle E_{pl}^{\text{int}} \rangle$) and the solvation free energy (ΔG_{sol}). All values are in kcal/mol. *The experimental value is -5.6 kcal/mol.

Time (1C5T)	AMBER					PPC				
	ΔH	$-T\Delta S$		ΔG_{bind}		ΔH	$-T\Delta S$		ΔG_{bind}	
		N_{mode}	IE	N_{mode}	IE		N_{mode}	IE	N_{mode}	IE
1	-36.09	18.95	27.70	-17.14	-8.39	-39.22	18.35	31.89	-20.87	-7.33
2	-35.06	16.70	27.70	-18.36	-7.36	-38.57	16.67	31.89	-21.90	-6.68
3	-35.34	15.82	27.70	-19.52	-7.64	-37.97	15.36	31.80	-22.61	-6.17
4	-35.02	13.42	27.69	-21.60	-7.33	-39.51	17.99	31.76	-21.52	-7.75
5	-36.32	16.18	27.69	-20.14	-8.63	-38.99	17.55	31.76	-21.44	-7.23
6	-34.57	16.76	27.70	-17.81	-6.87	-38.74	19.52	31.74	-19.22	-7.00
7	-37.54	19.08	27.69	-18.46	-9.85	-38.65	17.75	31.73	-20.90	-6.92
7	-36.47	16.45	27.70	-20.02	-8.77	-37.89	18.24	31.72	-19.65	-6.17
9	-36.61	17.17	27.71	-19.44	-8.90	-39.03	16.13	31.69	-22.90	-7.34
10	-37.34	18.35	27.74	-18.99	-9.60	-39.09	16.13	31.65	-22.96	-7.44
11	-36.25	16.81	27.76	-19.44	-8.49	-39.73	16.20	31.61	-23.53	-8.12
12	-34.92	16.10	27.79	-18.82	-7.13	-38.87	16.83	31.58	-22.04	-7.29
13	-35.11	19.67	27.82	-15.44	-7.29	-38.91	17.65	31.63	-21.26	-7.28
14	-36.32	17.53	27.86	-18.79	-8.46	-38.20	16.86	31.61	-21.34	-6.59
15	-37.29	16.00	27.87	-21.29	-9.42	-38.45	18.91	31.57	-19.54	-6.88
16	-34.56	16.65	27.85	-17.91	-6.71	-39.32	16.10	31.54	-23.22	-7.78
17	-35.13	17.78	27.87	-17.35	-7.26	-39.19	15.31	31.72	-23.88	-7.47
18	-35.71	17.91	27.86	-17.80	-7.85	-38.66	14.72	31.72	-23.94	-6.94
19	-35.45	17.00	27.87	-18.45	-7.58	-39.46	17.72	31.68	-21.74	-7.78
20	-36.31	17.22	27.87	-19.09	-8.44	-40.02	16.32	32.73	-23.70	-7.29
AVE	-35.87	17.08	27.77	-18.79	-8.10	-38.92	17.02	31.75	-21.91	-7.17
STD				1.42	0.93				1.44	0.52

Table S6 Binding free energy between trypsin and ligand in AMBER and PPC force field during the 20 additional 10 ns MD simulation for the 1O2J system. ΔH represents the total of protein–ligand interaction ($\langle E_{pl}^{\text{int}} \rangle$) and the solvation free energy (ΔG_{sol}). All values are in kcal/mol. *The experimental value is -7.8 kcal/mol.

Time (1O2J)	AMBER					PPC				
	ΔH	$-T\Delta S$		ΔG_{bind}		ΔH	$-T\Delta S$		ΔG_{bind}	
		N_{mode}	IE	N_{mode}	IE		N_{mode}	IE	N_{mode}	IE
1	-34.20	22.94	34.86	-11.26	0.66	-38.39	18.03	34.14	-20.36	-4.25
2	-34.30	20.62	34.88	-13.68	0.58	-40.54	20.71	34.14	-19.83	-6.40
3	-34.09	21.20	34.89	-12.89	0.80	-38.13	21.98	34.13	-16.15	-4.00
4	-33.77	20.19	34.91	-13.58	1.14	-38.21	19.14	34.13	-19.07	-4.08
5	-35.85	20.38	34.93	-15.47	-0.92	-37.20	19.11	34.13	-18.09	-3.07
6	-33.30	21.02	34.95	-12.28	1.65	-39.21	22.08	34.13	-17.13	-5.08
7	-30.97	23.06	34.95	-7.91	3.98	-39.90	22.15	34.15	-17.75	-5.75
8	-34.42	20.86	34.96	-13.56	0.54	-39.26	21.57	34.14	-17.69	-5.12
9	-34.01	24.01	34.97	-10.00	0.96	-40.55	24.78	34.15	-15.77	-6.40
10	-34.29	23.64	34.97	-10.65	0.68	-39.61	22.64	34.16	-16.97	-5.45
11	-34.32	22.59	34.96	-11.73	0.64	-40.17	22.86	34.20	-17.31	-5.97
12	-35.56	19.67	34.96	-15.89	-0.60	-42.40	23.59	34.23	-18.81	-8.17
13	-33.73	21.61	34.96	-12.12	1.23	-38.16	21.81	34.23	-16.35	-3.93
14	-33.55	22.90	34.96	-10.65	1.41	-37.08	20.74	34.22	-16.34	-2.86
15	-35.53	23.64	34.97	-11.89	-0.56	-37.09	20.53	34.20	-16.56	-2.89
16	-36.81	22.43	34.97	-14.38	-1.84	-36.95	19.39	34.18	-17.56	-2.77
17	-38.61	22.52	34.99	-16.09	-3.62	-39.39	20.51	34.19	-18.88	-5.20
18	-39.12	23.32	35.01	-15.80	-4.11	-39.29	23.79	34.23	-15.50	-5.06
19	-40.19	23.79	35.03	-16.40	-5.16	-39.88	22.37	34.26	-17.51	-5.62
20	-39.44	23.52	35.05	-15.92	-4.39	-36.57	21.45	34.27	-15.12	-2.30
AVE	-35.30	22.20	34.96	-13.11	-0.35	-38.90	21.46	34.18	-17.44	-4.72
STD				2.38	2.35				1.42	1.51

Fig. S1 The root-mean-square deviation (RMSD) of the backbone atoms relative to the corresponding native structure during the 200 ns MD simulation. The upper part of the figure is system 1C5T in AMBER (black) and PPC (red); and the lower part of the figure is system 1O2J.

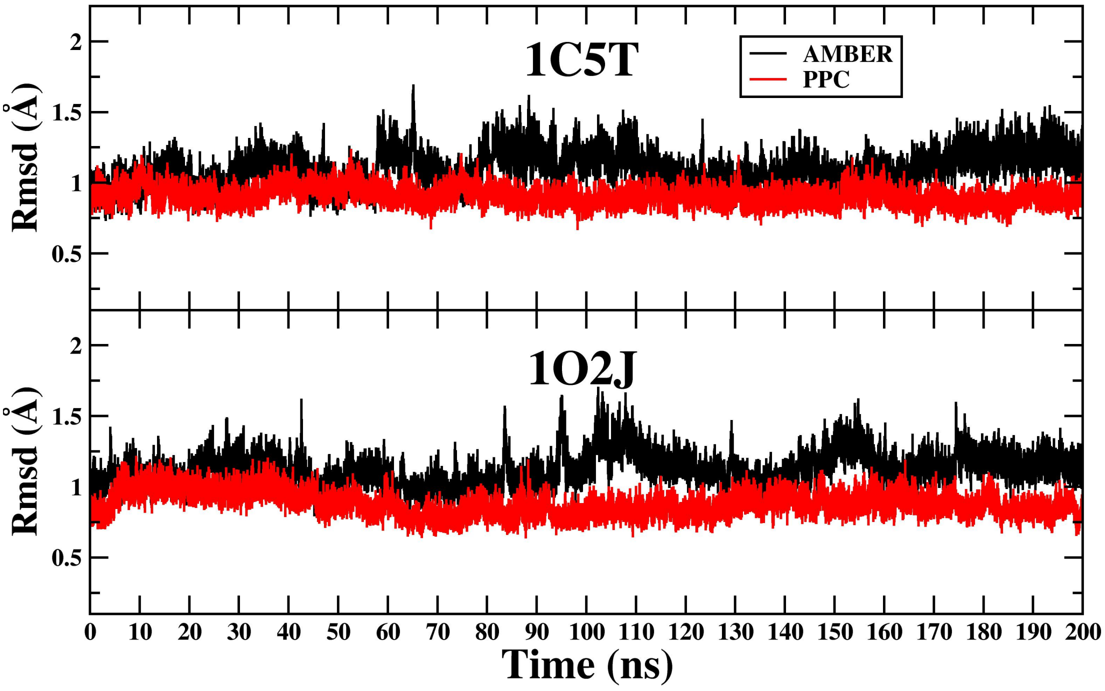

Fig. S2 The binding free energy distribution of 20 sets of results for 1C5T system calculated by IE and nmode method under PPC and AMBER.

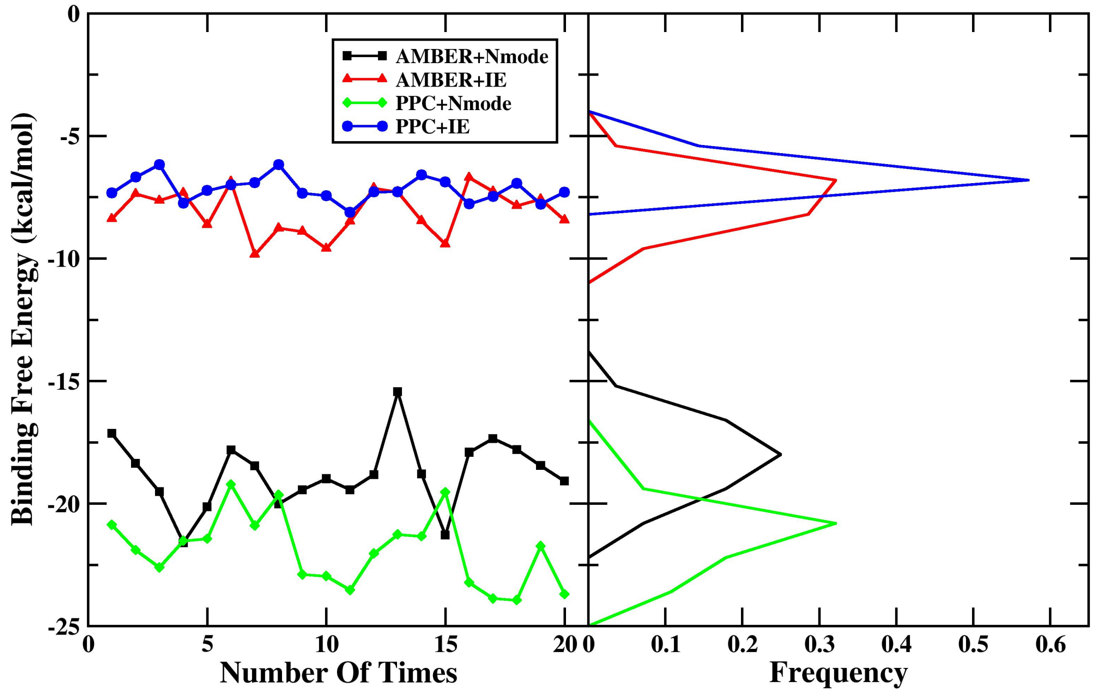

Fig. S3 The binding free energy distribution of 20 sets of results for 1O2J system calculated by IE and nmode method under PPC and AMBER.

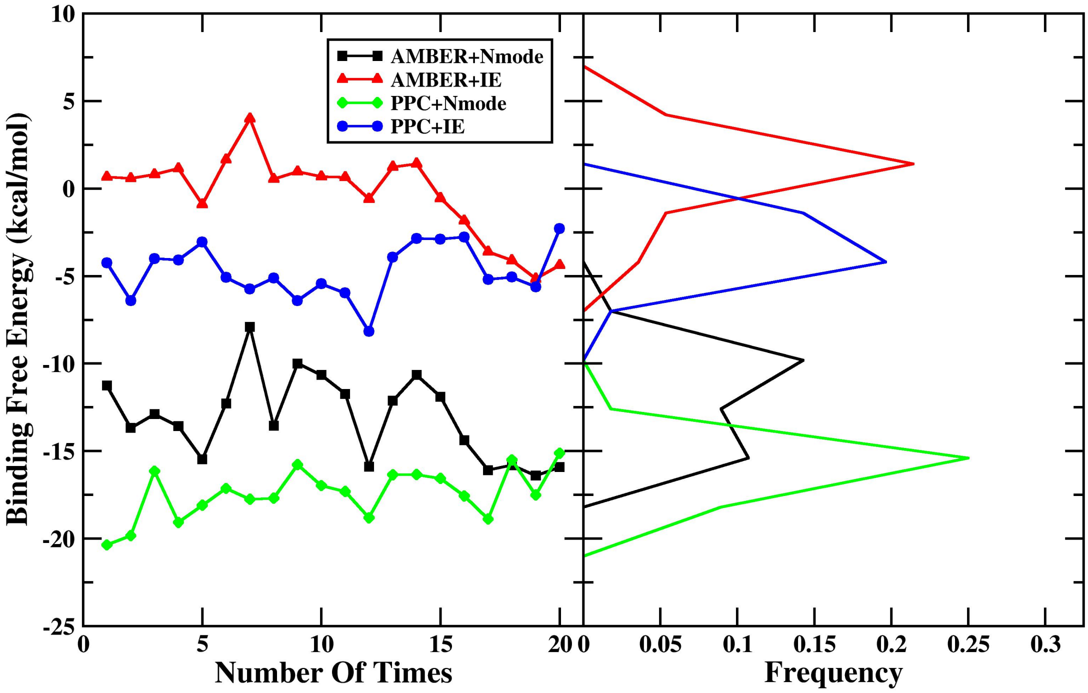

Supplement: Supplementary file 1 — supporting information [file 41598_2017_17868_MOESM1_ESM.pdf]
